# Pulmonary Adenofibroma: Clinicopathological and Genetic Analysis of 7 Cases With Literature Review

**DOI:** 10.3389/fonc.2021.667111

**Published:** 2021-07-19

**Authors:** Zuoyu Liang, Ping Zhou, Yuxuan Wang, Ying Zhang, Dan Li, Xiaoxing Su, Yu Fan, Yuan Tang, Lili Jiang, Weiya Wang

**Affiliations:** ^1^ Department of Pathology of West China Hospital, Sichuan University, Chengdu, China; ^2^ Department of Respiratory and Critical Care Medicine, Frontiers Science Center for Disease-Related Molecular Network, and Precision Medicine Center, Precision Medicine Key Laboratory of Sichuan Province, West China Hospital, Sichuan University, Chengdu, China; ^3^ Bioinformatics Department of Berry Oncology Corporation, Fuzhou, China

**Keywords:** pulmonary adenofibroma, fibroadenoma, immunohistochemistry, solitary fibrous tumor, pulmonary hamartoma

## Abstract

**Introduction:**

Pulmonary adenofibroma (PAF), characterized by biphasic differentiation composed of gland-like space lined by respiratory epithelium and stromal spindle cells, is a rare benign tumor of the lung. PAF was reported infrequently and inconsistently with diagnostic criteria and withstood higher risk of misdiagnosis as solitary fibrous tumors (SFTs) due to their morphological resemblance. In this study, we report seven cases of PAF with gene sequencing results and summarize the data of previous literature.

**Materials and Methods:**

Seven cases of PAF with surgically resection samples were collected from Pathology department of West China Hospital, Sichuan University between 2009 to 2020. Immunohistochemical studies were performed in all cases and 3 cases underwent a 425-gene panel next-generation sequencing (NGS).

**Results:**

Five female and two male patients were included in this study, with an average age of 51 years. All the patients were asymptomatic, and the lesion was identified on routine chest radiography. The tumor size measured by computed tomography (CT) ranged from 0.5 to 2.7 cm. Gland-like structures were mostly positive for glandular epithelium markers. The spindle cells in stroma expressed Desmin, SMA, ER and PR in 3 of 7 cases. No well-recognized molecular abnormalities can be identified by NGS in the 3 cases. To date, all the patients are alive, with no evidence of recurrence and metastasis.

**Conclusion:**

PAF is a unique benign pulmonary tumor with low incidence. Biphasic morphology, IHC stains along with molecular detection is of great significance to make a clear diagnosis.

## Introduction

Pulmonary adenofibroma is an extremely rare benign tumor, morphologically similar to fibroadenoma and phyllodes tumors of the breast. Since it was first described by Scarff in 1944 (1), approximately thirty cases of PAF had been reported worldwide. Pulmonary adenofibroma was not listed in the 2015 WHO Classification of Tumors of the Lung, Pleura, Thymus and Heart, probably due to its low incidence (2). Previous studies presented a confusing boundary between PAF and solitary fibrous tumors (SFT) (3–5).

Therefore, we collected 7 cases of PAF diagnosed in our department with a literature review, to investigate their clinicopathologic features along with genetic alterations of this rare tumor and compared them with SFT.

## Materials and Methods

### Patient Material

Seven formalin-fixed and paraffin-embedded (FFPE) samples of surgically resected pulmonary adenofibroma diagnosed from 2009 to 2020 in the Pathology Department of the West China Hospital, Sichuan University were collected, on the basis of morphology and related IHC staining. Every patient underwent surgical resection, with wedge resection in 5 patients and lobectomy in 2 patients. These specimens were reviewed by two experienced pathologists. Relevant data of all cases was collected as follows: Sex, age, smoking status, tumor size, location and follow-up. After obtaining institutional authorization and the approval from Ethics Committee, inform consent can be exempted in this retrospective observational research.

### Immunohistochemical Staining

Four-μm sections cut from representative available FFPE blocks of each case were evaluated for a panel of immunohistochemical markers, including Pan-CK (clone AE1/AE3, BIO), TTF-1 (clone 8G7G3/1, ZECA), EMA (clone GP1.4, BIO), ER (clone SP1, Roche), PR (clone 1E2, Roche), CD34 (clone EP88, BIO), STAT6 (clone, EP325, MXB), S-100 (clone 4C4.9, MXB), SMA (clone UMAB237, BIO), Desmin (clone MX046, MXB). The stain was performed on Leica Bond-Max or Roche Ventana system. The positive percentage and expression pattern (membrane, cytoplasm or nucleus) were evaluated. Staining results were regarded as positive when it expressed moderate or greater intensity in more than 10% of neoplastic cells. Reactivity less than 10% of neoplastic cells were considered as negative.

### Tissue and Plasma DNA Isolation and Purification

Genomic DNA (gDNA) was extracted from the sufficient and qualified FFPE samples using the GeneRead DNA FFPE Kit (Qiagen, Germantown, MD, USA) and from peripheral blood mononuclear cells using the DNA Blood Midi/Mini kit (Qiagen) according to the manufacturer’s instructions. Plasma cell-free DNA (cfDNA) was isolated using the MagMAX Cell-Free DNA Isolation Kit (Thermo, Waltham, MA, USA) according to the manufacturer’s protocol. The quality of purified DNA was assayed by gel electrophoresis and quantified with a Qubit 4.0 Fluorometer (Life Technologies, Carlsbad, CA, USA).

### Library Construction and ctDNA and Whole Exome Sequencing

Purified gDNA was first fragmented into DNA pieces of approximately 300 bp using an enzymatic method (5 × WGS Fragmentation Mix; Qiagen). After end-repairing and A tailing, T-adaptors were ligated on both ends, followed by PCR amplification to obtain a pre-library. The final sequencing libraries were prepared using the 96 rxn xGen Exome Research Panel v1.0 (Integrated DNA Technology, Coralville, IA, USA) according to the manufacturer’s protocol. For the targeted sequencing of cfDNA, the prelibraries were prepared according to the method previously described (6). In-house panels were designed to capture cfDNA fragments to generate sequencing libraries. The sequencing libraries were evaluated using the NovaSeq 6000 platform (Illumina, San Diego, CA, USA) in 150PE mode.

### Bioinformatics Analysis of Whole Exome Sequencing Results

The raw sequencing reads were subjected to quality control by trimming adaptor sequences and removing poly-N sequences (>10%) and low-quality reads (<Q20) preprocessed using FASTP (7). The FASTQ files were aligned to the human reference genome (hg19/GRCh37) using Burrows–Wheeler Aligner (BWA, v0.7.15) (8). Picard (2.12.1) (http://picard.sourceforge.net/) was used to process PCR duplicates for mapped BAM files. GATK (the Genome Analysis Toolkit 4.0.11.0) (9) was used for local realignment and base quality recalibration was employed to compute sequencing coverage and depth. Single nucleotide variants (SNVs) and small insertions and deletions (indels) were identified using GATK MuTect2. Mutations in the ENCODE Data Analysis Consortium blacklist were removed.

Variants were annotated using ANNOVAR (10) based on multiple databases, including HGVS variant description and population frequency databases (1000G, ExAC, and dbSNP), disease or phenotype databases (OMIM, COSMIC, and ClinVar), and variant functional in silico prediction tools (PolyPhen-2 and SIFT). After annotation, SNVs annotated as genomicSuperDups with a variant allele frequency (VAF) < 0.2 or PopFreqMax > 0.05 were excluded and nonsynonymous SNVs with a VAF > 3% or with a VAF > 1% in cancer hotspots collected from patient databases were retained for further analyses.

## Results

### Patient Characteristics

**Figure 1:**
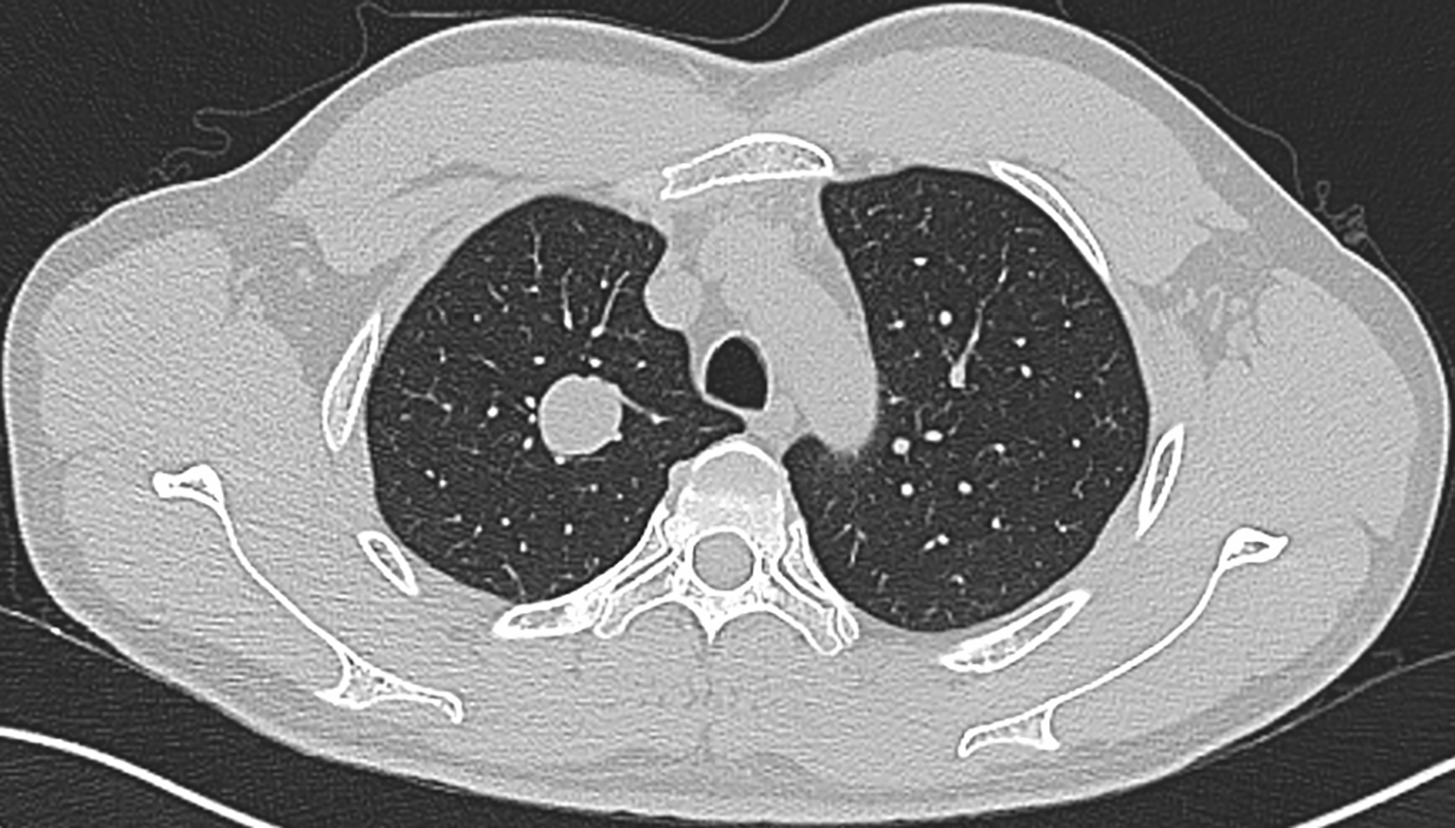
CT scanning of a case shows the lesion closely related to segmental bronchi.

Our series included 5 female and 2 male patients, with an average age of 51 years (range: 34 to 66 years) (**Table 1**). All the patients were asymptomatic, and the lesion was identified on routine radiography. CT images showed a solitary, well-circumscribed solid nodule or mass on the peripheral lung, with a maximum diameter ranging from 0.5 to 2.7 cm. Notably, one case occurred near the segmental bronchus, and the minimum distance from the pleura was 1.75 cm (**Figure 1**). A slightly lobulated pattern was observed in 2 cases. Three tumors were in the right upper lobe, 3 in the left lower lobe, and 1 in the left upper lobe. No obvious calcification, necrosis, inflammatory reaction or any malignant signs, such as a speculated margin or pleural traction, were observed in these tumors. Six patients were nonsmokers, except for one man who smoked for 30 years.

**Table 1:** The summary of clinical features of PAF in the literature and our cohort.

Author	Year	Case	Gender	Age	Size (cm)	Location		Follow up (after surgical operation)
S.SUSTER (11)	1993	2	M	56	2	left	upper	Died with myocardial infarction 5 years later
			F	54	1	right	upper	NED for 8 years
A Cavazza (3)	2003	1	M	62	0.8	right	lower	NED for 18 months
Yi Wang (12)	2012	1	M	55	2	left	lower	NED for 16 months
Taisia Vitkovski (13)	2012	1	F	29	3.5	left	upper	NED for 7 months
Rajiv Kumar (14)	2014	3	M	25	4.5	left	lower	NED for 5 years
			F	40	5	left	lower	NED for 1 year
			F	55	2.2	left	lower	NED for 4 months
Junmei Hao (15)	2016	1	F	57	1.5 (largest)	left (largest)	upper (largest)	NED for 11 months
Nicola Fusco (5)	2017	7	F	65	2.5	left	upper	NED for 9 months
			M	67	2	left	lower	NED for 17 months
			F	75	3	left	upper	NED for 12 months
			F	63	1.5	right	lower	NED for 9 months
			M	63	1.8	left	lower	NED for 22 months
			F	48	1.9	right	middle	NA
			M	74	0.5	right	lower	NED for 10 months
Mohammad Al-Amer (16)	2017	1	M	59	0.9	left	upper	NA
Nicholas J. Olson (17)	2018	1	M	60	1.7	left	lower	NED for 2 years
Kazuya Matsuda (18)	2018	1	–	**-**	2.5			–
Ramona Erber (19)	2020	1	NA	64	NA	right	lower	NA
Kaleigh E. Lindholm (20)	2020	13	8F	41 - 73	1 - 2.5	5 left	6 upper	NED for 12 to 36 months
			5M			8 right	7 lower
			F	54	2.3	left	lower	NED for 11 years
			M	62	2.5	right	upper	NED for 17 months
			F	46	0.5	right	upper	NED for 7 months
Our data		7	F	47	1.2	left	upper	NED for 1 year
			F	66	1	left	lower	NED for 5 years
			F	45	2.7	left	lower	NED for 5 months
			M	34	2	right	upper	NED for 3 months
Total		40	22F	25 -75	0.5 - 4.5	22 left	17 upper	
			16M			17 right	21 lower	

NA, not available; NED, no evidence of disease recurrence.

### Pathological Features

**Figure 2:**
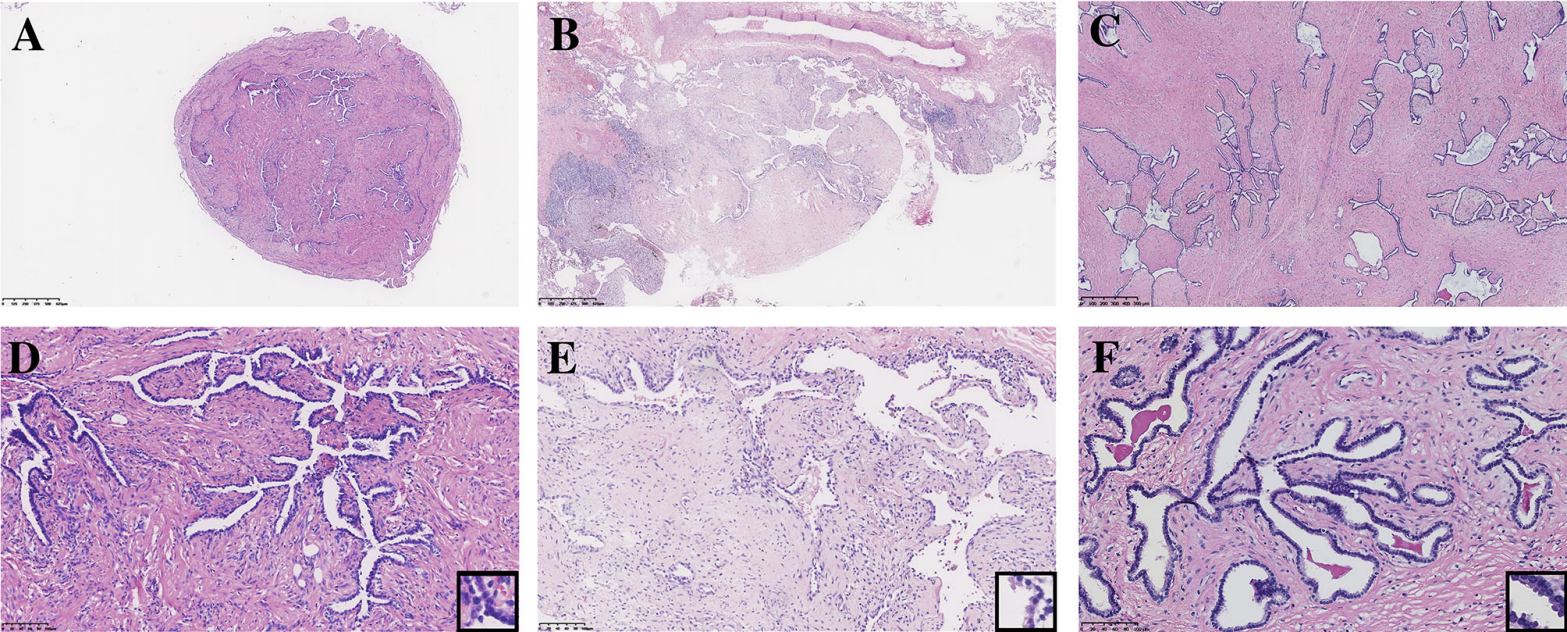
Typical morphology of three PAFs [H.E. staining, **(A–C)** x40, **(D–F)** x200, inset x400]. Focal fat vacuoles **(D)** and intraglandular eosinophilic fluid **(F)** were seen.

**Figure 3:**
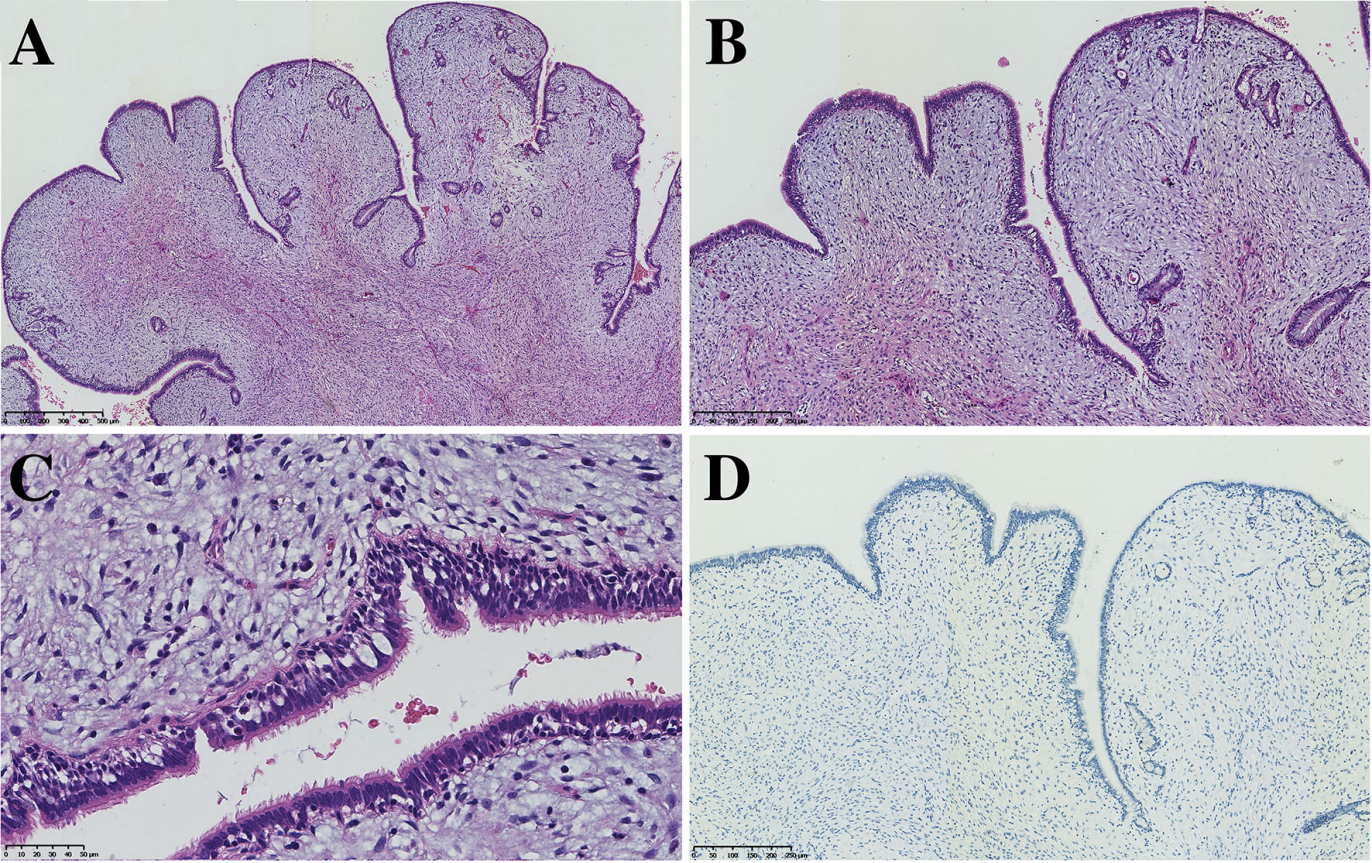
PAF can also harbor the respiratory epithelium of large bronchi (**A**, x50, **B** x100 **C** x200) without expression of TTF-1 (**D**, x100).

All seven PAF tissues presented a gray and solid appearance. Histologically, the lesions consisted of glandular lumens and interstitial components. The glandular lumens were lined with simple squamous, cuboidal or columnar epithelium, stratified squamous epithelium and other distal bronchus or alveolar epitheliums in five cases (**Figure 2**). Clear cytoplasm could be seen in the cuboidal epithelium. Additionally, diffuse pseudostratified ciliated columnar epithelium and interspersed goblet cells were observed (**Figures 3A–C**) in the case which the tumor was located near the segmental bronchus. The stroma is comprised of diffuse spindle cells and collagen matrix. The respiratory epithelium is devoid of atypia and mitotic activity. Squeezed by the stromal compartment, the glandular lumens presented a tubular structure or even slit-like space and frequently contained erythrocytes, macrophages and eosinophilic fluid. One PAF showed minimal fat vacuoles near the epithelium (**Figure 2D**), and calcification was found in another case. No evidence of cartilage, hemorrhage or necrosis was found in our cases. Two cases presented an unclear margin microscopically, while the other five cases were well circumscribed. Four cases showed a leaf-like or phyllodes-like growth pattern in the peripheral part of the tumor.

Immunohistochemistry staining showed that PCK and EMA were positive in the glandular epithelium of all PAFs, while TTF-1 showed no expression in 2 cases (**Figure 3D**). For stromal spindle cells, 3 cases expressed ER, PR, SMA and Desmin simultaneously, and CD34 was positive in 3 of 7 cases (**Table 2**). Tumor cells of all detected cases were negative for STAT6 and S-100.

**Table 2:** Integrating the immunophenotypes of pulmonary adenofibroma.

Author	Year	Case	Epithelium			Stroma									
			CK	TTF-1	EMA	ER	PR	BCL-2	CD34	STAT6	S-100	SMA	Desmin	Vimentin	CD99
S.SUSTER (11)	1993	2	NA	NA	NA	NA	NA	NA	NA	NA	–	NA	–	+	NA
											–		–	+	
A Cavazza (3)	2003	1	+	+	+	NA	NA	focal+	+	NA	–	–	–	+	focal+
Yi Wang (12)	2012	1	+	+	+	NA	NA	NA	+	NA	NA	NA	NA	+	NA
Taisia Vitkovski (13)	2012	1	+	+	NA	NA	NA	–	–	NA	focal+	weak+	+	+	NA
Rajiv Kumar (14)	2014	3	+	+	+	NA	NA	–	+	NA	–	focal+	focal+	+	NA
			+	+	NA	NA	NA	NA	NA	NA	NA	focal+	NA	NA	NA
			+	+	NA	NA	NA	NA	NA	NA	NA	focal+	NA	NA	NA
Junmei Hao (15)	2016	1	+	+	NA	+	+	+	–	NA	NA	+	+	+	–
Nicola Fusco (5)	2017	7	+	+	NA	+	+	+	+	+	NA	NA	NA	+	+
			+	+	NA	–	–	+	+	+	NA	NA	NA	+	+
			+	+	NA	+	+	+	+	+	NA	NA	NA	+	+
			+	+	NA	–	focal+	+	+	+	NA	NA	NA	+	+
			+	+	NA	+	+	+	+	–	NA	NA	NA	+	+
			+	+	NA	+	+	+	+	–	NA	NA	NA	+	+
			+	+	NA	–	–	+	+	+	NA	NA	NA	+	+
Mohammad Al-Amer (16)	2017	1	NA	NA	NA	NA	NA	NA	NA	NA	NA	–	–	NA	NA
Nicholas J. Olson (17)	2018	1	+	+	+	+	+	NA	+	–	NA	NA	NA	NA	NA
Kazuya Matsuda (18)	2018	1	+	+	NA	NA	NA	NA	NA	NA	–	+	+	+	NA
Ramona Erber (19)	2020	1	NA	+	NA	–	–	NA	NA	–	NA	+	NA	NA	NA
Kaleigh E. Lindholm (20)	2020	13	13+	NA	NA	NA	NA	13-	13-	13-	13-	13-	13-	13+	NA
			+	+	+	NA	NA	NA	–	NA	–	–	–	NA	NA
			+	+	+	–	–	NA	–	–	–	–	–	NA	NA
			+	+	NA	+	+	NA	+	–	NA	+	+	NA	NA
Our data		7	NA	+	+	+	–	+	–	–	NA	–	–	NA	NA
			+	–	+	–	–	NA	+	–	NA	–	–	NA	NA
			+	+	+	+	+	+	+	–	–	+	+	NA	NA
			+	–	NA	+	+	NA	–	–	–	+	+	NA	NA
Total		40	35+	22+	9+	10+	10+	11+	14+	5+	1+	10+	7+	28+	8+
				2-		6-	6-	15-	19-	23-	22-	19-	21-		1-

Abbreviations as in **Table 1**.

### Molecular Alteration

Next-generation sequencing was performed in 4 samples from 3 patients (Case 2, 3, 4), which included one additional pulmonary invasive adenocarcinoma sample from patient (Case 4) who present with coexisting adenocarcinoma with PAF in a same lobe.

Eleven to fourteen mutated genes were identified in each tumor tissue of these 3 PAFs, and none was reported to be related to malignant tumorigenesis. Mutations of *JAK1* and *PTPN11* (*SHP2*) coexisted in one patient. No patients had more than 3 splicing mutations, but all mutations were located on different chromosomes and had no correlation. The other genetic abnormalities were point mutations. Two patients had 3 sites of mutations in the *MUC17* gene, and the other patients shared no mutations in the same gene. Sixteen mutated genes were found in the adenocarcinoma tissue, which contained the driver gene *HER2* mutation. However, the mutated genes found in adenocarcinoma were different from those genes of PAF lesion in the same patient.

### Treatment and Follow-Up

After the follow-up of these 7 patients from 4 months to 11 years, no evidence of tumor recurrence or metastatic disease was found.

## Discussion

The understanding of the PAF progressed fairly slow on account of its rare incidence. First, PAF was described as a subtype belonging to the same tumor family as pulmonary hamartomas (1, 21). After that, the distinction between PAF and SFT was ambiguous due to morphological similarity. PAF was described as a special variant of SFT in some studies (3, 4), especially in one patient that PAF and SFT coincidentally occurred in the same lobe of the lung (17). In recent years, most scholars have supported that PAF belongs to an independent entity, while more cases and further studies are still needed to investigate the mechanism of tumorigenesis.

The detailed data of these 7 patients and cases in the literature are summarized in **Table 1**. The clinicopathological features of our cases were consistent with those of patients in the literature. Gender preference showed that PAF was more common in female than male patients. For predilection sites, the tumors were located slightly more in the left lung than in the right lung, with no significant difference between the upper and lower lobes. Most PAFs were asymptomatic and detected by physical examination, while some can presented with cough (14, 16, 20), chest pain (12, 13, 20) and breathlessness (13, 16, 20). PAF radiographically presented as a solitary, well-circumscribed solid nodule on the peripheral lung, consistent with the features of benign tumors. The maximum diameters mostly ranged from 0.5 to 4.5 cm (5, 14, 20). Some studies found that PAF grew very slowly, with no signs of malignant transformation during the 7 years of follow-up (12).

PAF is histologically heterogeneous. Typical cases are characterized by epithelial and stromal components: stromal spindle fibroblastic or myofibroblastic cells and glandular elements lined by slightly respiratory epithelium (some show a leaf-like epithelial pattern). Eosinophilic secretions can sometimes be found in the glandular lumina (14). Epithelial cells with a clear cytoplasm are frequently present (13), indicating that they may secrete glycogen or mucus. Calcification and myxoid changes could occasionally be seen, but no intratumoral hemorrhage or necrosis are reported (14, 20).

The neoplastic component of PAF remains controversial. Most scholars considered PAF as a biphasic tumor, composed of neoplastic epithelium and stromal spindle cells (14, 18–20). Other scholars argued that the glandular component of PAF is entrapped normal respiratory epithelium during tumor growth and only stroma is genuine neoplastic component (1, 15). Fusco et al. even thought that epithelium of PAF is bronchiolar metaplasia based on the expression of E-cardherin, TTF-1 and its ultrastructural features (5).

Regarding immunohistochemical studies, CK, EMA and TTF-1 were widely expressed in the epithelium, while the expression of mesenchymal components was different among different cases. Previous studies have described PAF as a peripheral lesion, and TTF-1 was invariably positive. In our series, however, two TTF-1-negative cases with the tumor located away from the pleura suggest that epithelium is derived from a larger bronchus. Only a few cases showed positive expression of Bcl-2 (11/26), CD34 (14/33), and S-100 (1/23) in the spindle cells of the stroma. Desmin (7/29) and SMA (10/29) were expressed in some PAFs, likely indicating an immunophenotype of myofibroblasts in those spindle cells.

In recent years, ER and PR were found to be occasionally positive in the stroma, possibly indicating that PAF is potentially hormone related (5, 20). Two female patients and one male patient in our series were positive for ER, PR, SMA and Desmin simultaneously, which does not support this point of view. No pelvis or breast mass was found in these 2 female patients, excluding the possibility of metastatic smooth muscle tumors of the uterus or malignant phyllodes tumors of the breast. What’s more, no statistically significant differences were found in the expression of hormone receptors between different sexes in all the cases.

Fusco et al. reported that 5 of 7 PAFs were nuclear positive for STAT6 protein. In their study, sequencing results also showed that 4 of 7 cases, similar to SFT cases, harbored the specific *NAB2ex4-STAT6ex2* fusion, indicating that PAF has a similar genetic origin as SFT (5). Olson et al. even reported that a 60-year-old male patient developed PAF and SFT in the same lung. Next-generation sequencing was performed in these two lesions, but the *NAB2-STAT6* fusion was only identified in SFT (17). Several large-scale studies on soft tissue tumors showed that nuclear positivity for the STAT6 antibody and *STAT6* fusion gene were mostly characterized by SFT (22–25). Lindholm et al. noted that STAT6 protein was negative in their 13 PAFs, and the expression of CD34 and Bcl-2 was also significantly different between SFT and PAF (20). Given their findings, Lindholm et al. considered PAF as an independent entity that must be separated from SFT. All the cases in our series were negative for STAT6 protein. Combined with the negative sequencing results, we also agreed that using IHC staining or molecular detection to exclude SFT is essential to demonstrate diagnosis of PAF.

Currently, NGS has only been performed in one PAF in the literature (17). Hence, we performed NGS on 3 cases of PAF tissues together with one adenocarcinoma tissue coincidently occurring in the same lobe of one patient (Case 4) to further clarify its molecular characteristics. The results showed that PAF did not carry the *NAB2-STAT6* gene fusion in SFT or the genetic changes in breast fibroepithelial tumors, such as *MED2*, *RARA*, and *FLNA* (26). The mutations detected in PAF were unrelated to the tumorigenesis of malignant tumors, and relevant studies were lacking. *MUC17* mutated with SNVs or indels at three codon sites was detected in two PAF cases. The *MUC17* gene is located on chromosome 7q22. RNA blotting and *in situ* hybridization proved that it could be expressed in intestinal absorption cells, colon cancer and pancreatic cancer cells (27), while corresponding research in the lung tissue was scant. Located on the surface of the cell membrane, the family of membrane mucins execute several crucial functions, including cytoprotection (28), migration promotion (29) and signal transduction (30, 31). Mutation of the *MUC17* gene may also lead to abnormal function of goblet cells, which are widely arranged on the bronchial epithelium.

For the patient with PAF and adenocarcinoma in the same lobe, SNVs of *HER2* were detected in adenocarcinoma, and SNVs of *JAK1* and *PTPN11* genes were found in PAF, suggesting that PAF is unlikely to be related to the occurrence of adenocarcinoma. As a collateral branch of the JAK-STAT pathway and upstream of the MAPK signaling pathway, *JAK1* and *PTPN11* are related to cell proliferation, differentiation and transformation (32).

In addition to SFT, PAF should be differentiated from other benign and malignant tumors. More than two mesenchymal components can be found in hamartoma, such as cartilage, smooth muscle, adipose tissue, loose mucus-like tissue or bone. In particular, when clear cartilage or obvious fat tissue is present in the stroma, the diagnosis of PAF should be excluded. Sclerosing pneumocytoma (SP) has similar gender preference, tumor size and even macroscopic appearance compared to PAF. SP typically demonstrates a more varied growth pattern consisting of papillary, sclerotic, and solid growth as well as comprising two cell types (2). The extensive existence of monomorphic round cells in stroma with immunoactivity of TTF-1 and epithelial membrane antigen is crucial to differentiate from PAF (33, 34). Papillary adenoma consists of papillary structures containing fibrovascular cores lined by a single layer of cuboidal epithelium, which is different from PAF (2). Of note, papillary adenoma is thought to have low malignant potential for its occasionally infiltrative growth (35–37). However, there are no reported cases of metastatic disease or recurrence in PAF, and the tumor is generally considered to be benign and is cured by resection. Alveolar adenomas usually form multiple microscopical spaces lined by type II pneumocytes, which is filled with finely granular proteinaceous material (2). Glandular structure accompany with fibroblasts proliferation can be seen in some alveolar adenomas (38). Compared to PAF, stressed lumen and prominent spindle cells in stroma are generally scant in alveolar adenoma. Mesothelioma can present with adenoid structures, but it is closely related to the pleura and expresses mesothelial markers (e.g. calretinin, D2-40, and WT-1) in the tumor components. The epithelial component of biphasic synovial sarcoma can be characterized as a slit-like or tube-like glandular cavity, while the epithelial component does not express TTF-1 or Napsin A. Additionally, the positivity of TLE-1 protein and translocation of the *SS18* gene are helpful for the differential diagnosis. Sarcomatoid carcinoma, particularly pulmonary blastoma and carcinosarcoma with bidirectional differentiation, has apparent dysplasia and mitotic activity, which can be easily distinguished from the glandular epithelium with bland morphology in PAF. Metastatic breast phyllodes tumors can also show phyllodes structures, but they can be differentiated based on ER, PR, TTF-1 and Napsin A staining and clinical history. Given that various lung tumors can more or less mimic the morphological pattern of PAF, Erber et al. commended that the exceptionally rare genuine PAF should be considered an exclusive diagnosis (19).

In summary, as a rare benign pulmonary lesion, PAF has not been listed in the 2015 WHO Classification of Tumors of the Lung, Pleura, Thymus and Heart. Pathologists should be aware of its unique morphology, and a definite diagnosis should be confirmed by immunohistochemical staining and even genetic detection. Additionally, clinicians should be familiar with its indolent biological behavior and excellent prognosis to avoid overtreatment. Consequently, more cases and further researches are needed to further illustrate its clinicopathological characteristics.

## Data Availability Statement

The datasets presented in this study can be found in online repositories. The names of the repository/repositories and accession number(s) can be found below: DNA Data Bank of Japan (DDBJ) and BioSample accession(s): SAMD00281797-SAMD00281800 Temporary Submission ID: SSUB017358.

## Author Contributions

WW conceived and designed the study. ZL contributed to write the text, create the table and figure. YF revised the manuscript. All authors contributed to the article and approved the submitted version.

## Conflict of Interest

Author YF was employed by company Berry Oncology Corporation.

The remaining authors declare that the research was conducted in the absence of any commercial or financial relationships that could be construed as a potential conflict of interest.
